# Too Many Mouldy Joints – Marijuana and Chronic Pulmonary Aspergillosis

**DOI:** 10.4084/MJHID.2011.005

**Published:** 2011-01-14

**Authors:** Yousef Gargani, Paul Bishop, David W. Denning

**Affiliations:** 1 The National Aspergillosis Centre, University Hospital of South Manchester, The University of Manchester, Manchester Academic Health Science Centre, and School of Medicine. Manchester, United Kingdom; 2 Department of Histopathology, University Hospital of South Manchester, The University of Manchester, Manchester Academic Health Science Centre, and School of Medicine. Manchester, United Kingdom

## Abstract

Chronic pulmonary aspergillosis is a progressive debilitating disease with multiple underlying pulmonary diseases described. Here we report the association of chronic pulmonary aspergillosis and long term marijuana smoking in 2 patients and review the literature related to invasive and allergic aspergillosis.

## Introduction

Marijuana is the most commonly used illicit substance in the UK and many other western countries, despite it being a class B drug. It is available legally in some localities, including the Netherlands. There is a well known link between marijuana use and schizophrenia. There is currently uncertainty about any causal association between marijuana use and lung cancer as the effects of concomitant tobacco smoking amongst these users confound analyses. A small number of cases of various forms of aspergillosis have been associated with marijuana smoking, but the association appears to be uncommon.^1^–^9^

We present 2 cases of chronic pulmonary aspergillosis (CPA) associated with extensive medicinal use of marijuana, and summarise the literature linking all forms of aspergillosis and marijuana use.

## Case reports

### Patient 1

**Figure 1:**
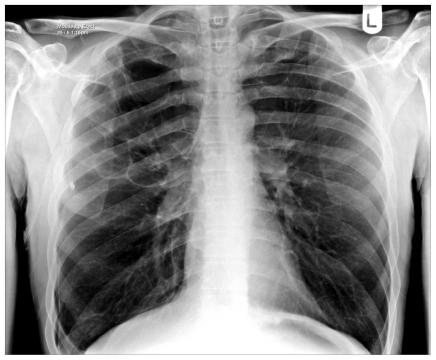
Patient 1. Bilateral apical bullae, more marked on the right; associated with a right pneumothorax.

A Caucasian male presented at the age of 47 with a right-sided pneumothorax (Figure 1), associated with pulmonary bullae. He had a four year history of progressive breathlessness. His tobacco smoking history was approximately 39 pack*years, but his breathlessness worsened considerably once he started to smoke marijuana (5 marijuana cigarettes (joints) daily) medicinally to alleviate rheumatoid arthritis-associated joint pain. On presentation, he reported coughing up thick sputum and experiencing some unexpected weight loss. His medications included a 5mg daily dose of prednisolone and 1g dose of sulfasalazine twice a day. His family history included one brother who had TB and another who had a pneumothorax.

**Figure 2:**
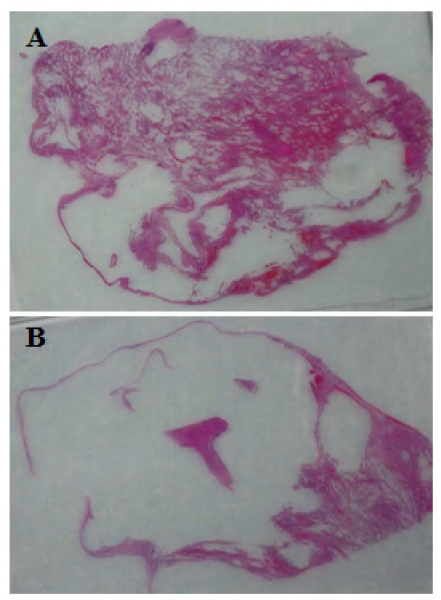
Appearance of the resected lung showing remarkable loss of normal lung architecture, and large airspaces, especially in section B.

**Figure 3:**
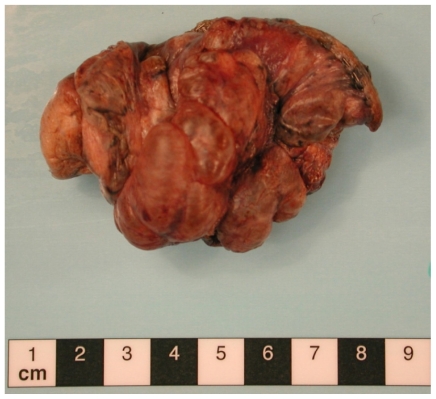
Appearance of the fungal ball found at surgery.

**Figure 4:**
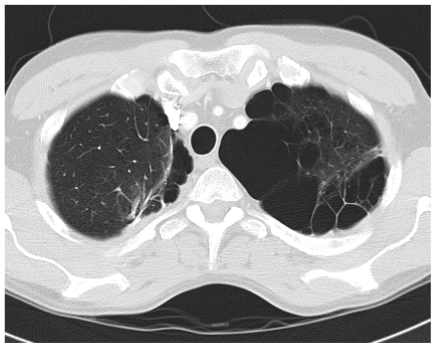
Patient 1 Postoperative CT thorax showing many large lung bullae in left apex with smaller bullae, and a bullectomy suture line in the right. The aspergilloma and surrounding cavity that was found has been excised.

His pneumothorax did not resolve despite drainage; thus he underwent a right bullectomy and pleurectomy. One of the excised bullae (Figure 2) contained a pleural based abscess containing an aspergilloma (Figure 3). His CRP at this time was 333mg/l. Postoperatively his lung function failed to improve and a chest x-ray revealed that that his right lung remained abnormal, with cavitary changes. A CT scan showed severe emphysema with many large lung bullae especially in the left apex (Figure 4). His CRP normalised and his alpha 1 antitrypsin levels were normal.

**Figure 5:**
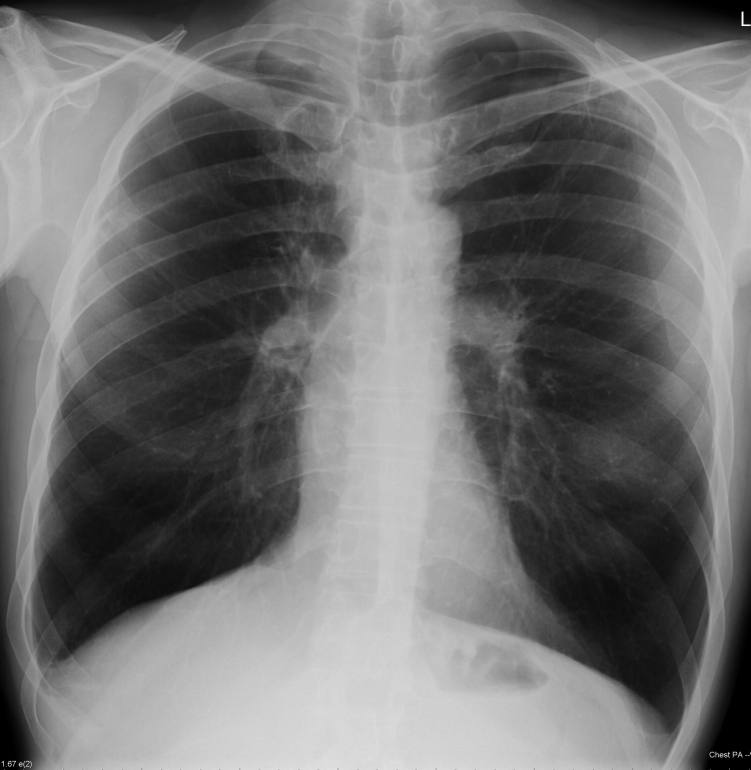
Patient 1 Chest radiograph 3 years later, showing generalised emphysema but no recurrence of chronic pulmonary aspergillosis

Pathological evidence of an aspergilloma with symptoms of weight loss and raised inflammatory markers is an indication for antifungal therapy. He was started on posaconazole 400mg bd and took it for 4 months. After treatment both his cough and sputum production improved and his *Aspergillus fumigatus* precipitin serum titre was 1:4. He stopped smoking marijuana. He was observed for recurrence of CPA for the following 4 years and there was no evidence of recurrence (Figure 5).

### Patient 2

A 35 year-old Caucasian male presented with shortness of breath secondary to a viral infection. After investigations he was diagnosed with emphysema. He had been born with a Tetralogy of Fallot congenital heart malformation and underwent a surgical repair at the age of 10. Post-operatively he experienced immense pain and started smoking cannabis, without tobacco, to reduce it. He continued to grow his own marijuana and smoke several joints daily.

He remained apparently well until admission to hospital aged 43, and then several times over the following two years, owing to type II respiratory failure. Aged 44, he stopped smoking cannabis,ending 34 years of smoking around 20 joints per day. Despitestopping smoking cannabis his respiratory function had diminished to such an extent that his exercise tolerance was only 10 yards on the flat. As a result he was started on long term oxygen therapy, requiring 3L continuously to maintain an oxygen saturation of 91%.

**Figure 7:**
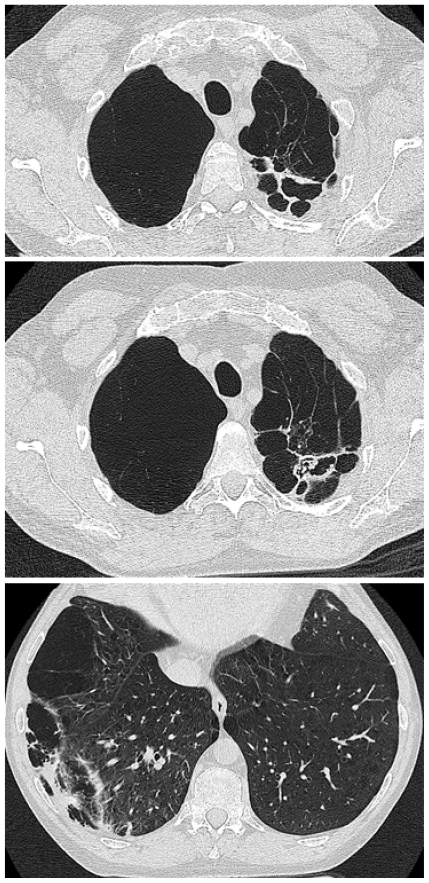
Patient 2 CT scan cuts of the thorax showing extensive bullae formation on the right, cavitation posteriorly on the left, with fungal material present in a cavity on the second section, and additional areas of cavitation in the right lower lobe, with slightly thicker walls and pleural involvement.

By 45 years his FEV_1_ was only 11% predicted and his FVC was 22% predicted. A CT scan revealed “severe pan-acinar emphysema involving the right upper and middle lobes; complex cavitary lesions in the left apex, one of which had the appearance of an aspergilloma” (Figure 7). Sputum culture revealed *Aspergillus fumigatus*. His *Aspergillus* precipitins titre was 1:8 and his *Aspergillus* IgG antibody level (Immunocap, Phadia) was 126mg/L. His total IgE was 180 and he had a positive *Aspergillus* RAST of 0.4, all consistent with chronic pulmonary aspergillosis. His respiratory function deteriorated such that he required a lung transplant; however the discovery of an aspergilloma was seen to be an absolute contraindication by the team treating him. He was started on voriconazole 200mg bd with some symptomatic improvement on therapeutic levels. However his lung function continued to deteriorate and 3 months later he died at the age of 46.

## Discussion

*Aspergillus* spp. are widespread and exposure to airborne conidia and hyphal fragments is universal. In humans who are immunocompromised these saprophytic fungi can cause life-threatening invasive aspergillosis and, in asthmatics and those with cystic fibrosis, allergic disease (allergic bronchopulmonary aspergillosis (ABPA)). In those who have structural damage to their lungs but are apparently immunocompetent, *Aspergillus* can cause CPA, with or without an aspergilloma.

Invasive aspergillosis has been described in association with marijuana smoking in two cancer patients on chemotherapy,^1^,^2^ two leukaemia patients,^3^,^4^ a renal transplant recipient,^5^ and a few patients with AIDS.^6^ It is not recommended that patients undergoing chemotherapy or on substantial immunosuppression smoke marijuana, based on these observations. However the risk is not possible to quantify.

There have been two reported cases of ABPA associated with mouldy marijuana.^7^,^8^ Given the frequency of asthma and ABPA, and the high frequency of marijuana usage amongst young people, these cases may represent a tiny proportion of those affected. This year, CPA was associated with marijuana smoking for the first time^9^ and we report here 2 additional cases. The previously reported patient died at just 34 years of age and patient 2 at 46 years of age. These high mortality rates of CPA have been described previously.^10^–^12^

The criteria for the diagnosis of CPA are the presence of pulmonary cavitation, with or without a fungal ball, in a non-immunocompromised patient with evidence of raised inflammatory markers and detectable circulating *Aspergillus* IgG antibodies (including *Aspergillus* precipitins).^13^ The sensitivity of the IgG antibody tests is about 90%, with some discordance between assays. Occasional patients do not have detectable IgG antibody, but their Aspergillus IgE antibodies are raised and/or there are other data supporting the diagnosis, such as biopsy or culture evidence of infection. Patient 1 had histological evidence of infection with detectable *Aspergillus* precipitins. Patient 2 had a positive respiratory culture, with a fungal ball visible radiologically, and detectable Aspergillus IgG and IgE antibodies, as well as positive Aspergillus precipitins.

Both tobacco and marijuana are commonly contaminated with fungi, and serology from marijuana smokers exhibits evidence of Aspergillus exposure.^14^ It remains to be seen whether fungal spores survive the burning process, indicating perhaps that exposure to Aspergillus comes from handling the marijuana rather than smoking it.^15^ It has been observed that IA presents earlier after immunocompromise amongst smokers than non-smokers.^16^

The way marijuana is smoked is different to that of tobacco in that the cigarettes, or joints, are usually smoked without a filter and are smoked down to a smaller butt. Users hold their breath for longer and use the Valsalva manoeuvre. These measures maximise the diffusion of the psychoactive compounds. In doing so however, they expose their lungs to a greater tar and carbon monoxide burden, as well as subjecting their lungs to greater pressure changes. This barotrauma from marijuana smoking has long been associated with the formation of bullae and subsequent pneumothoraces. The association is so strong that some now recommend that patients presenting with spontaneous pneumothoraces should be directly questioned about marijuana smoking.^17^

The vast majority of marijuana is taken recreationally for its psychotropic effects. However, historically it has been used medicinally as an analgesic and an antiemetic. Even now, many people use marijuana purely for its medicinal benefits. It has been reported that 44% of US oncologists have recommended the illegal use of marijuana to their chemotherapy patients for iatrogenic nausea.^18^ What must be borne in mind is that these patients are potentially severely immunosuppressed and therefore at risk of life-threatening IA.

Smoking marijuana affects the lungs structurally but may also affect them immunologically, by affecting alveolar macrophages.^19^ This may predispose marijuana users to pulmonary infection. In our cases it is unknown whether the marijuana smoking caused the cavities which *Aspergillus* was able to colonise, or the marijuana was just the source of *Aspergillus* exposure and the *Aspergillus* caused the cavities. Research into the pathogenesis of both marijuana-related lung bullae and CPA are needed to proportionate causal blame in cases such as these.

Marijuana use has previously been implicated with the range of *Aspergillus* infections; the cases we present add aspergilloma and CPA to that list. In both cases marijuana was used for its medicinal benefits. With patient 1 smoking marijuana in combination with mild immunosuppression through low-dose steroids proved to be a potent combination. In patient 2 the heavy and prolonged marijuana usage in itself may have resulted in extensive pulmonary destruction and fatal CPA.

Together these cases highlight yet another potential risk associated with smoking marijuana. Understanding the full health burden presented by marijuana is hindered by a lack of disclosure by patients for fear of legal repercussions, coupled with poor history taking from clinicians; just 3% of daily marijuana smokers have their drug use documented in their clinical record.^20^ More work is required to elucidate the full health burden of marijuana on those with pre-existing pathologies, so that governments, clinicians, and indeed users can make informed decisions about this controversial topic.

## Figures and Tables

**Figure 6:**
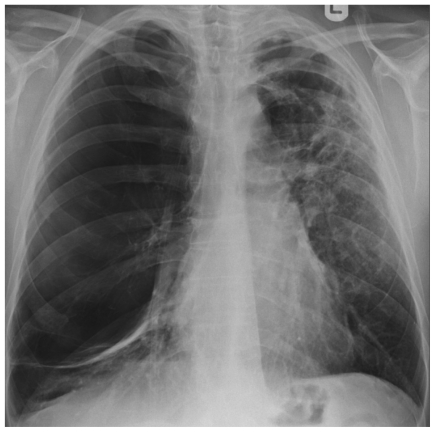
Patient 2 Chest radiograph, showing extensive loss of lung architecture, 2 curvilinear opacities in the left base and the upper left mediastinum representing compressed lung from adjacent bullae formation. On the left side there is parenchymal shadowing and the impression of thin walled cavities just above the hilum.
